# A Transient Transgenic RNAi Strategy for Rapid Characterization of Gene Function during Embryonic Development

**DOI:** 10.1371/journal.pone.0014375

**Published:** 2010-12-16

**Authors:** Bryan C. Bjork, Yuko Fujiwara, Shannon W. Davis, Haiyan Qiu, Thomas L. Saunders, Peter Sandy, Stuart Orkin, Sally A. Camper, David R. Beier

**Affiliations:** 1 Genetics Division, Brigham & Women's Hospital, Harvard Medical School, Boston, Massachusetts, United States of America; 2 Division of Hematology and Oncology, Children's Hospital, Harvard Medical School/Howard Hughes Medical Institute, Boston, Massachusetts, United States of America; 3 Departments of Human Genetics and Internal Medicine, University of Michigan, Ann Arbor, Michigan, United States of America; 4 The David H. Koch Institute for Integrative Cancer Research, Massachusetts Institute of Technology, Cambridge, Massachusetts, United States of America; University of Birmingham, United Kingdom

## Abstract

RNA interference (RNAi) is a powerful strategy for studying the phenotypic consequences of reduced gene expression levels in model systems. To develop a method for the rapid characterization of the developmental consequences of gene dysregulation, we tested the use of RNAi for “transient transgenic” knockdown of mRNA in mouse embryos. These methods included lentiviral infection as well as transposition using the *Sleeping Beauty* (*SB*) and *PiggyBac* (*PB*) transposable element systems. This approach can be useful for phenotypic validation of putative mutant loci, as we demonstrate by confirming that knockdown of *Prdm16* phenocopies the ENU-induced cleft palate (CP) mutant, *csp1*. This strategy is attractive as an alternative to gene targeting in embryonic stem cells, as it is simple and yields phenotypic information in a matter of weeks. Of the three methodologies tested, the *PB* transposon system produced high numbers of transgenic embryos with the expected phenotype, demonstrating its utility as a screening method.

## Introduction

The production of targeted mutations in mice remains the gold standard for the analysis of the loss-of-function studies of specific genes in mammals. However, even with the emergence of large-scale knockout mouse resources, such as those of the International Knockout Mouse Consortium (http://www.knockoutmouse.org/), generation of such mutants using embryonic stem (ES) cells may still require substantial time and resources. In particular, this approach is difficult to pursue for high throughput applications. For instance, linkage and association studies for mutations or strain-specific traits may yield a large number of positional candidate genes, which may require testing individually to assess causality. Similarly, microarray analyses typically result in lists of differentially expressed genes, with little indication regarding which ones may be key regulators. An efficient methodology to rapidly screen genes *in vivo* would enhance the functional analysis of outputs from high throughput screening.

The discovery of RNA interference (RNAi) and its application in mammals has provided a new avenue to study the consequences of reduced gene expression [1], [2]. In this process, short 19–25 nt double-stranded RNA (dsRNA) duplexes mediate the degradation of mRNA transcripts that contain an exact match to the dsRNA sequence (reviewed in [3]). This occurs through the recruitment of the RNase III enzyme, Dicer, followed by a multicomponent nuclease complex known as RISC (RNA-induced silencing complex). Alternatively, mismatched dsRNAs can lead to reduced gene activity through the suppression of protein translation [4].

Current methods for the utilization of RNAi as a means to test the effect of loss of gene function involve direct introduction of short interfering RNAs (siRNAs) or expression of precursor short hairpin RNAs (shRNAs) expressed on plasmids and retroviruses [2], [5], [6]. shRNA-expressing vector systems, including lentivirus and transposable elements vectors, provide highly efficient, stable shRNA expression in cultured cells and transgenic mammals (reviewed in [7], [8]). Lentiviral infection of ES cells, morula, or single-cell embryos (via injection into the perivitelline space) has been successfully employed for transgenesis in mice and subsequent RNAi knockdown [9], [10]. However, these protocols are not routinely employed in microinjection facilities. In contrast, the *Sleeping Beauty (SB)* and *PiggyBac (PB)* transposon systems can be employed using standard microinjection protocols that yield substantially higher transgenic efficiency than traditional pronuclear DNA injections [7], [11], [12], [13 and this study]. These transposon systems have two-components, the first of which is a transposon vector containing an expression cassette flanked by terminal inverted repeats that have binding sites for the *SB or PB* transposase in direct orientation, termed IR/DRs. The second component is *SB or PB* transposase mRNA, which can be co-expressed from a plasmid or transcribed *in vitro*. The specific transposase mediates transposition via a “cut and paste” mechanism in which the transposable element is excised from a donor plasmid, followed by its integration into the host genome at a specific target DNA sequence: TA for *SB*; TTAA for *PB*. *SB* transposons have recently been used in combination with RNAi to achieve stable reduction of gene expression in cultured cells [14].

One of several potential applications of a rapid method for RNAi knockdown in embryos is the validation of N-ethyl-N-nitrosourea (ENU)-induced mutations. ENU screens performed in mice have successfully identified a wide spectrum of abnormal phenotypes affecting development [15], [16], [17], [18]. The mutations induced by ENU can affect non-coding regulatory sequences and will not be discovered by the usual exon-directed sequencing analysis. In addition, it is possible that multiple ENU-induced mutations are present within the genetically defined recombinant interval carrying the causal locus. Therefore, even when a putative mutation is identified, independent validation of the positionally-cloned gene mutation is desirable. We explored whether RNAi could be efficiently used for targeted mutagenesis by employing a “transient transgenic” protocol; i.e., transgenic analysis in which microinjected embryos are not used to generate stable lines, but rather examined directly. Similar approaches to assay loss of gene function have been used successfully in zebrafish [19], [20].

**Figure 1:**
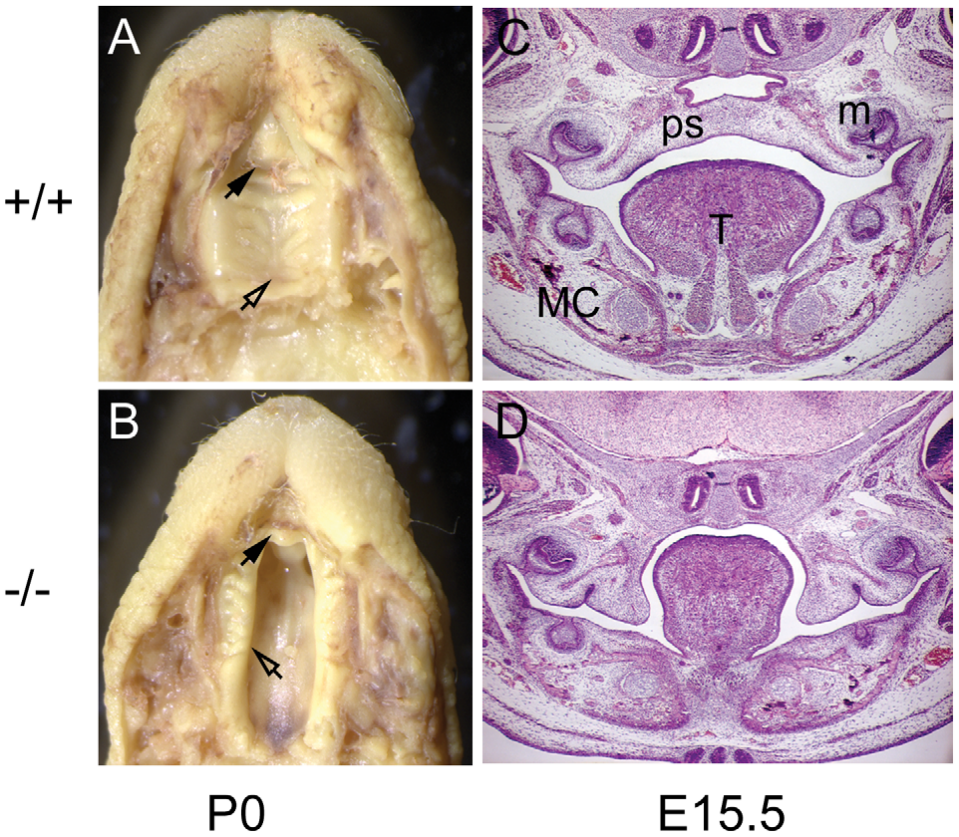
The recessive ENU-induced *csp1* mutation in *Prdm16* exhibits cleft secondary palate. Homozygous *csp1* mutants are born with cleft palate (CP) and die within 24 hours after birth (**A–D**). Bouin's fixed wild type (**A**) and *csp1* (B) mutant newborn pups with CP (**A** and **B**). The unaffected primary palates (black arrowheads) and fused or cleft secondary palate are evident (open arrowheads in **A** and **B**, respectively). Hematoxylin and Eosin-stained coronal sections through wild-type (**C**) and *csp1* mutant (**D**) embryonic day (E) 15.5 embryos show impaired palate shelf elevation in mutants. Tongue, T; palate shelf, ps; Meckel's cartilage, mc and molar, m.

We have previously identified the *cleft secondary palate 1* (*csp1*) mutant in an ENU mutagenesis screen for recessive late-term developmental anomalies that model human birth defects [15]. Newborn homozygous *csp1* mutant pups on an FVB/NJ strain background exhibit cleft secondary palate with virtually complete penetrance and die within 24 hours (Fig. 1A and B). Positional cloning revealed that this mutant carries an intronic splicing mutation in the *Prdm16* zinc finger transcription factor gene on distal chromosome 4. We have since confirmed the etiology of the *csp1* mutation in *Prdm16* by carrying out a complementation test with a *Prdm16* gene trap mutation [21].

In this study, we utilized several variants of lentivirus and *SB* and *PB* transposons to express *Prdm16*-specific shRNAs and compared their efficacy for transgenesis and phenotypic validation of the mutant allele. RNAi knockdown of *Prdm16* using each system successfully recapitulated the *csp1* CP phenotype in transient transgenic mouse embryos. Lentiviral infection yielded high transgenic efficiency with modest phenotypic penetrance. *SB* transposon-mediated transgenesis resulted in low transgenic efficiency with high phenotypic penetrance. However, nonviral *PB* transposon-mediated transgenesis yielded both high transgenic efficiency and high phenotypic penetrance. As this system is amenable for use in any laboratory and transgenic facility, it represents an ideal means for the rapid analysis of the consequences of mRNA knockdown in a mammalian system.

## Results

In mice, secondary palate development begins with palate shelf outgrowth from the maxillary prominences at E12.5, followed by downward growth along either side of the tongue and then concurrent rapid shelf elevation and flattening of the tongue at approximately E14. Fusion occurs between the medial edge epithelium (MEE) of the two palate shelves through a combination of epithelial-mesenchymal transformation, cell migration and apoptosis [22], [23]. Apposition and fusion of the palatal shelves at the midline occurs by E14.5 in most mouse strains. We initiated transient transgenic RNAi experiments in mice to examine the effect of reduced *Prdm16* expression in E16.5 mouse embryos, by which time wild type palate shelves have elevated and fused [23].

### Selection of efficient *Prdm16*-specific shRNAs for RNAi

**Figure 2:**
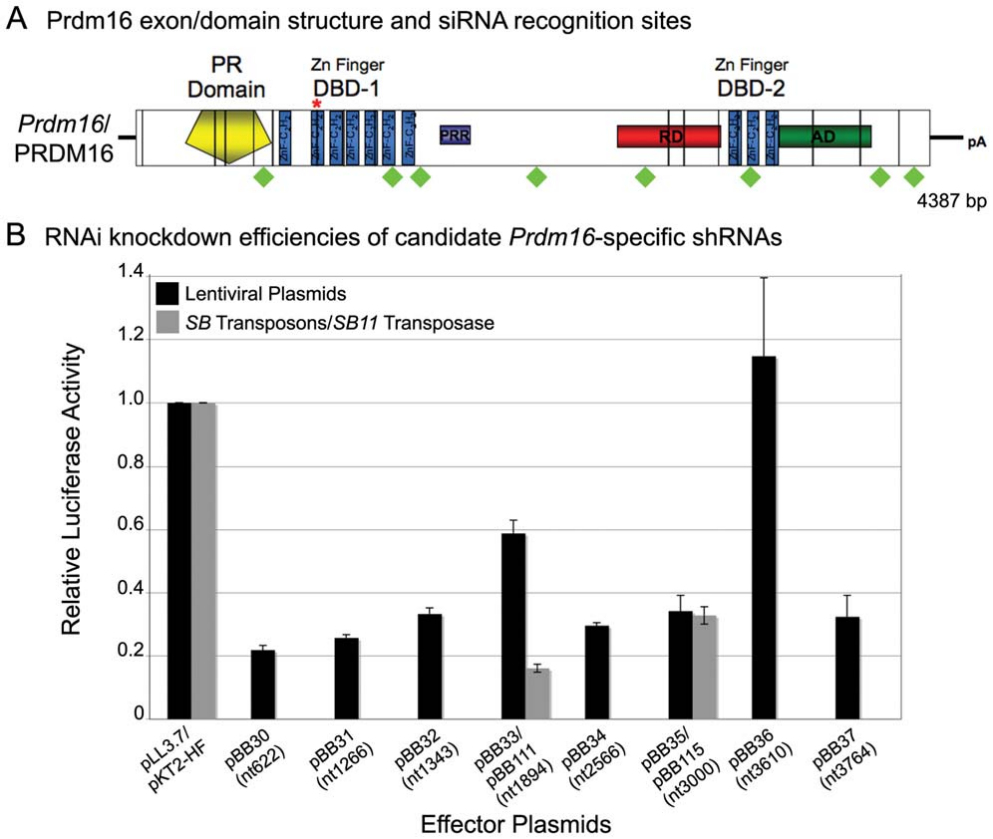
*Prdm16*-specific shRNA selection and validation of RNAi knock down efficiency. Schematic of *Prdm16* mRNA and protein structure (**A**). Vertical black lines demarcate exon boundaries. Conserved functional domains include a Positive Regulatory (PR) domain, two multi-fingered zinc finger DNA binding domains (DBD-1 and DBD-2), repressor domain (RD), acidic domain (AD) and Proline-rich region (PRR). Eight *Prdm16*-specific shRNAs are shown with respect to their positions within the *Prdm16* coding sequence (green diamonds). The red inverted “V” depicts the alternatively spliced exon 16. *Prdm16* mRNA knock down efficiency *in vitro* mediated by expression of the candidate shRNAs from pLL3.7 lentivirus plasmids or CpG-free *Sleeping Beauty (SB)* transposons measured by relative luciferase activity (**B**). RNAi knock down values for effector shRNAs were normalized against the knockdown efficiency of an empty plasmid control transfection, and transfection efficiencies were calculated based upon the co-transfection of a *Renilla luciferase* control expression plasmid. Transfections were performed in duplicate for screening purposes, and error bars show the standard deviations.

**Figure 3:**
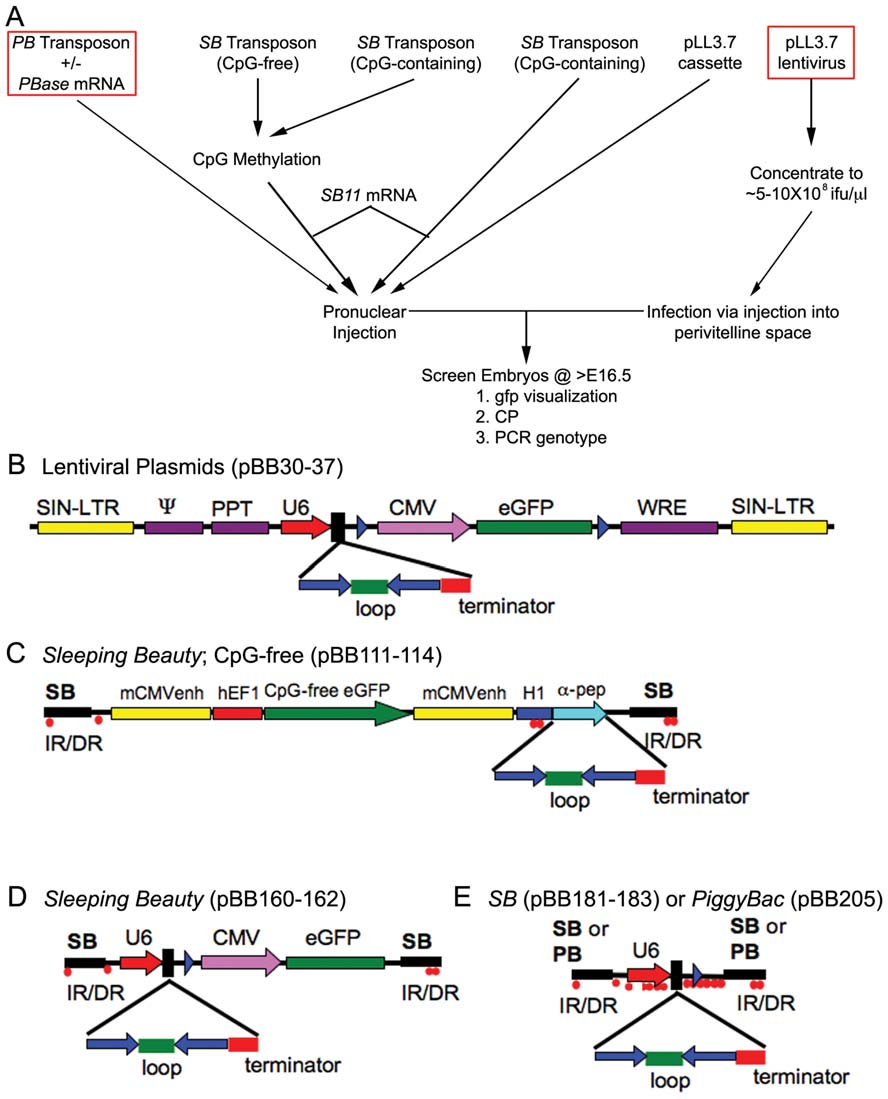
Strategy for gene mutation validation and candidate gene screening using transient transgenic RNAi knockdown. Flow chart outlining the experimental method and the classes of transgenic delivery vehicles and their variants (**A**). Creation of various shRNA-expressing lentivirus and *Sleeping Beauty* (*SB*) and *PiggyBac* (*PB*) transposon plasmids (**B–E**). **B**) pLL3.7 lentivirus plasmid was described previously and contains a U6–shRNA; CMV-eGFP expression cassette [10]. SIN-LTR, self-inactivating long terminal repeat; Ψ, HIV packaging signal; cPPT, central polypurine track; MCS, multiple cloning site; CMV, cytomegalovirus promoter; WRE, woodchuck hepatitis virus response element. Sense and antisense sequences that form the stem of the stem loop shRNA sequence are shown by the solid blue arrows; the loop sequence, green bar and the terminator, red bar. **C**) CpG-free EF1-GFP; H1-shRNA *SB* transposons. IR/DR, inverted/direct terminal repeats recognized by *SB* transposase; mCMVenh, mouse cytomegalovirus enhancer sequence; hEF1, human EF1 promoter; eGFP, synthetic *GFP* coding sequence; H1, human pol III promoter; α-pep, lacZ alpha peptide for blue-white selection. **D**) CpG-containing U6-shRNA; CMV-eGFP from pLL3.7 in the *SB* transposon. **E**) U6-shRNA expression cassette from pLL3.7 in the *SB* and *PB* transposons. CpG dinucleotides methylated by SssI methylase (red dots) in *SB* transposon experiments are shown.

*Prdm16* is comprised of 17 exons, and the *Prdm16* transcript is 4394 nucleotides and contains an open reading frame that encodes a 1277 amino acid protein (Fig. 2A, NM_027504) [24]. To identify a sequence that would mediate effective RNAi, we selected eight *Prdm16*-specific siRNA sequences that meet eight criteria previously associated with efficient siRNA knockdown (Table 1, Fig. 2A) [25]. We utilized sense and antisense shRNA oligonucleotides comprised of the sense siRNA target sequence, a stem loop sequence, the antisense siRNA target sequence, a 5-thymidine terminator sequence and appropriate overhangs for cloning (Table 1) [10]. Annealed sense and antisense shRNA oligonucleotides were ligated downstream of the human U6 small nuclear RNA polymerase III promoter in the lentiviral vector, pLentiLox3.7 (pLL3.7; Fig. 3B), which also contains a CMV-eGFP expression cassette for visualization of infected cells [10].

**Table 1:** *Prdm16*-specific shRNA expression plasmid sequences.

Plasmid #	Name	Plasmid backbone	Position of siRNA in cds	Sequence (5′-3′)
30	nt622	pLL3.7	684–702	F:TGTTGGTGCATGTGAAAGAATTCAAGAGATTCTTTCACATGCACCAACTTTTTTC
				R:TCGAGAAAAAAGTTGGTGCATGTGAAAGAATCTCTTGAATTCTTTCACATGCACCAACA
31	nt1266	pLL3.7	1328–1346	F:TGGACGCAGATCAAGTGCAATTCAAGAGATTGCACTTGATCTGCGTCCTTTTTTC
				R:TCGAGAAAAAAGGACGCAGATCAAGTGCAATCTCTTGAATTGCACTTGATCTGCGTCCA
32	nt1343	pLL3.7	1405–1423	F:TGAGGGCAAGAACCATTACATTCAAGAGATGTAATGGTTCTTGCCCTCTTTTTTC
				R:TCGAGAAAAAAGAGGGCAAGAACCATTACATCTCTTGAATGTAATGGTTCTTGCCCTCA
33	nt1894	pLL3.7	1956–1974	F:TGGACAGTGACAGAGACAAATTCAAGAGATTTGTCTCTGTCACTGTCCTTTTTTC
				R:TCGAGAAAAAAGGACAGTGACAGAGACAAATCTCTTGAATTTGTCTCTGTCACTGTCCA
34	nt2566	pLL3.7	2628–2646	F:TGCATTATGCTAAGCCTTCATTCAAGAGATGAAGGCTTAGCATAATGCTTTTTTC
				R:TCGAGAAAAAAGCATTATGCTAAGCCTTCATCTCTTGAATGAAGGCTTAGCATAATGCA
35	nt3000	pLL3.7	3065–3083	F:TGGAACATCCACAACAAAGATTCAAGAGATCTTTGTTGTGGATGTTCCTTTTTTC
				R:TCGAGAAAAAAGGAACATCCACAACAAAGATCTCTTGAATCTTTGTTGTGGATGTTCCA
36	nt3610	pLL3.7	3675–3693	F:TGGAAGCATTTGAAGTTAAATTCAAGAGATTTAACTTCAAATGCTTCCTTTTTTC
				R:TCGAGAAAAAAGGAAGCATTTGAAGTTAAATCTCTTGAATTTAACTTCAAATGCTTCCA
37	nt3764	pLL3.7	3829–3847	F:TGATGCTTGGTTGAACATCATTCAAGAGATGATGTTCAACCAAGCATCTTTTTTC
				R:TCGAGAAAAAAGATGCTTGGTTGAACATCATCTCTTGAATGATGTTCAACCAAGCATCA
111	nt1894	pKT2-HF (*SB*)	1956–1974	F:ACCTCGGACAGTGACAGAGACAAATTCAAGAGATTTGTCTCTGTCACTGTCCTTTTTTTT
				R:CAAAAAAAAAAAGGACAGTGACAGAGACAAATCTCTTGAATTTGTCTCTGTCACTGTCCG
113	nt622	pKT2-HF (*SB*)	684–702	F:ACCTCGTTGGTGCATGTGAAAGAATTCAAGAGATTCTTTCACATGCACCAACTTTTTTTT
				R:CAAAAAAAAAAAGTTGGTGCATGTGAAAGAATCTCTTGAATTCTTTCACATGCACCAACG
114	nt622-scrambled	pKT2-HF (SB)	NA	F:ACCTCGCGGAGAAAGTGGATTTATTTCAAGAGAATAAATCCACTTTCTCCGCTTTTTTTT
				R:CAAAAAAAAAAAGCGGAGAAAGTGGATTTATTCTCTTGAAATAAATCCACTTTCTCCGCG
160	Empty U6-no shRNA; CMV-eGFP	pKT2-HF (SB)	NA	None
161	U6-nt1266 shRNA; CMV-eGFP	pKT2-HF (SB)	1328–1346	Same as pBB31
162	U6-nt1894 shRNA; CMV-eGFP	pKT2-HF (SB)	1956–1974	Same as pBB33
181	Empty U6-no shRNA	pKT2-HF (SB)	NA	None
182	U6-nt1266 shRNA	pKT2-HF (SB)	1328–1346	Same as pBB31
183	U6-nt1894 shRNA	pKT2-HF (SB)	1956–1974	Same as pBB33
205	U6-nt622 shRNA	pCyL50 (PB)	684–702	Same as pBB30

Underlined nucleotides in each oligonucleotide sequence represent the sense and antisense sequence-specific siRNA target sequence within *Prdm16*.

We used a *luciferase* reporter system to assay the effectiveness of the shRNAs. The coding sequence for a splice variant of *Prdm16* in which exon 16 is absent was subcloned into the 3′ UTR of the *luciferase* gene contained on a modified pGL3 *Firefly luciferase* reporter plasmid (pGL3-DEST-*Prdm16*) [26]. To measure knockdown efficiency, luciferase activity was measured after co-transfection into 293T cells of each shRNA-expressing lentivirus plasmid with pGL3-DEST-*Prdm16* and normalized to the activity obtained from a co-transfected pRL-TK *Renilla luciferase* plasmid. All shRNA lentiviral plasmids showed knockdown activity, except for pBB36 (nt 3610), which is in the exon that is not included in the pGL3-DEST-*Prdm16* reporter plasmid (Fig. 2B). pBB30 (nt 622) and pBB31 (nt 1266) facilitated the strongest knockdown, to approximately 20% of wild-type expression levels. Transfection of *Prdm16*-specific shRNA-expressing *SB* transposon plasmids (pBB111, nt1894; pBB115, nt3000) also facilitated strong knockdown activity in this assay (Fig. 2B).

### Lentivirus and *Sleeping Beauty*/*PiggyBac* transposons expressing *Prdm16*-specific shRNAs recapitulate the *csp1* mutant CP phenotype in transient transgenic mouse embryos

To assay the developmental consequences of RNAi knockdown of *Prdm16* in mice, we performed “transient” transgenic analysis in which *Prdm16*-specific shRNAs were introduced into mouse embryos and litters were examined at E16.5 for the presence of CP and co-expression of *GFP* (Fig. 3A). We utilized lentivirus and *SB* or *PB* transposons (Fig. 3B-E) and assayed variables including shRNA knockdown efficiency, transgene delivery vehicle, transposon methylation status and size and presence or absence of the *GFP* reporter. For plasmid DNA injection and lentiviral infection, we used the pBB30, pBB33 and empty pLL3.7 plasmids described above. For *SB* transposon-mediated delivery of *Prdm16*-specific shRNAs, methylated or unmethylated transposon DNA and *in vitro*-transcribed 5′capped *Sleeping Beauty* transposase (*SB11*) mRNA [27] was injected into single cell embryos [11], [12]. Similarly, a *PB* transposon expressing a *Prdm16*-specific shRNA was co-injected with 5′-capped *PiggyBac* transposase *(PBase)* mRNA. A summary of all transient transgenic RNAi experiments is provided in Table 2.

**Table 2:** Combined summary of transgenic RNAi injections.

Constructs	Methylation	# Embryos	TG	CP	TG Frequency	CP Frequency	Penetrance	CP, not TG
Lentivirus plasmid	No	88	16	0	0.18	0	NA	0
empty								
U6; *GFP*								
Lentivirus plasmids	No	248	8	0*	0.03	0*	0*	0
nt1266, nt1894								
U6; *GFP*								
Lentivirus plasmid	No	55	26	5*	0.47	0.09	0.15	1
nt622								
U6; *GFP*								
*SB*	Yes	56	32	0	0.57	0	0	0
nt622 scrambled								
CpG-free H1; *GFP*								
*SB*	Yes	79	54	0	0.68	0	0	0
nt622								
CpG-free H1; *GFP*								
*SB*	No	32	4	0	0.13	0	0	0
Empty								
U6; *GFP*								
*SB*	No	108	5	4	0.05	0.04	0.8	0
nt1266, nt1894								
U6; *GFP*								
*SB*	No	57	4	3*	0.07	0.05	0.50*	1
nt1266								
U6; no *GFP*								
*SB*	Yes	73	12	2	0.16$	0.03	0.17$	0
nt1266								
U6; no *GFP*								
*PB*	No	59	10	1	0.17	0.02	0.10	0
nt622								
U6; no *GFP*								
No *PBase* control								
*PB*; nt622	No	112	52	12	0.46	0.11	0.23	0
nt622								
U6; no *GFP*								
*PBase*								
*PB*	No	20	2	1	0.10	0.05	0.50	0
nt622								
U6; no *GFP*								
*4-5X PBase*								

*Denotes one or more transgenic embryos showing early embryonic growth arrest prior to palate fusion.

$ Three of 24 resorptions were transgenic.

*TG*, transgenic embryos; *CP*, cleft palate; *Penetrance*, number of transgenic embryos with CP.

**Figure 4:**
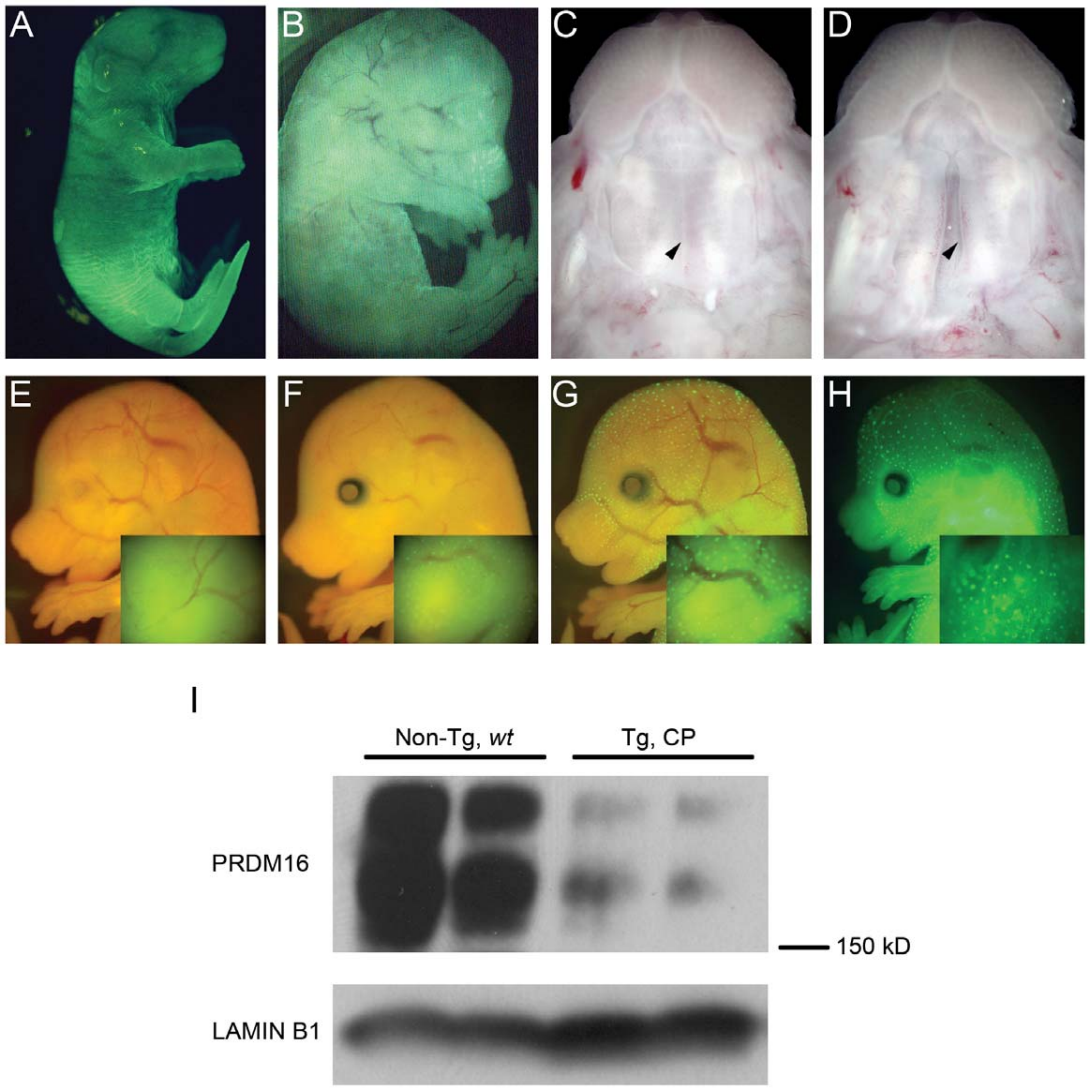
Transient transgenic RNAi knockdown of *Prdm16* in mice recapitulates the recessive *csp1* ENU mutant phenotype. A) Strong *GFP* expression driven by the CMV-eGFP cassette with plasmid DNA injection of pLL3.7. **B**) Similarly strong ubiquitous *GFP* expression visible in some lentivirus infected transient transgenic *Prdm16* RNAi knock down E16.5 embryos. Wild-type (**C**) and transgenic (**D**) E16.5 embryos with fused and cleft palate, respectively, representative of the CP phenotype observed in affected transgenic embryos produced using delivery vehicles reported in this study. Variable *GFP* expression pattern observed in transgenic embryos carrying CpG-free *SB* transposons with the H1-GFP cassette, also representative of the variability of *GFP* expression pattern observed in all constructs utilizing a *GFP* expression cassette (**E–H**). Insets consist of higher magnification images taken of head regions from these same embryos. **I**) Western blot analysis for PRDM16 in nuclear lysates isolated from non-transgenic wild type (Non-Tg, *wt*) and transgenic CP E16.5 embryonic heads derived from pronuclear injection of a *Prdm16*-specific shRNA-expressing *PB* transposon. LAMIN B1 expression is provided as a loading control.

To begin we used traditional transgenic methodologies for injection of pLL3.7, pBB30 and pBB33 plasmid DNA into single FVB/J mouse cells (Table 2). Control pLL3.7 injections yielded 18% transgenic newborn pups with strong, ubiquitous *GFP* expression (Fig. 4A), but pBB30 and pBB33 yielded substantially less transgenic embryos and none with CP. To produce transgenic embryos with increased frequency, we first performed transient transgenic experiments by lentiviral infection. High titer (∼0.5–1.0×10^9^ ifu/ml) lentivirus derived from pBB30 was injected into the perivitelline space of single FVB/NJ mouse oocytes (Table 2). Transgenic efficiency was 47% (26/55), but *GFP* expression was visible in only 7% (4/55) of embryos (Fig. 4B). 16% (4/26) of transgenic embryos exhibited CP (Fig. 4D compared to wild type embryo in Fig. 4C), with an additional embryo arresting prior to palatogenesis. One CP embryo was not transgenic by PCR genotyping. Lentiviral transgenesis proved to be an efficient strategy to validate the *csp1* mouse mutation, but the specialized training, certification and facilities required for lentivirus experiments, as well as the non-trivial task of isolating high titer lentivirus, may discourage the routine use of this strategy.

The nonviral *SB* and *PB* transposon systems have the potential to efficiently generate transient transgenic embryos expressing gene-specific shRNAs. Since CpG methylation of *SB* transposons can improve transposition frequency [28], [29], we first used the SssI methylase-treated CpG-free *SB* transposons and *SB11* mRNA for transient transgenic experiments (Fig. 3C, Table 2). We achieved high transgenic efficiency, 57% and 68%, with a control transposon (pBB114, scrambled nt622 shRNA) and pBB113 (nt622), respectively, accompanied by variable *GFP* marker expression (Fig. 4E-H). However, we observed no embryos with CP upon dissection at E15.5 (Table 2).

Therefore, we altered several variables in an attempt to improve shRNA and *GFP* expression. First, we used the U6 Pol III promoter, which drives shRNA expression with greater efficiency than the H1 Pol III promoter [30]. We subcloned the CpG-containing U6-shRNA; CMV-eGFP expression cassette (1.94 kb) from pLL3.7 into the *SB* transposon plasmid. *GFP* expression is robust when expressed from this cassette *in vivo* (Fig. 4A). We did not methylate these transposons before injection due to the presence of many CpG dinucleotides (Fig. 3A, data not shown). Transgenic injection of pBB160 (control), pBB161 (nt1266) and pBB162 (nt1894) produced much lower transgenic efficiencies than with the CpG-free transposons, (13%, 5% and 5%, respectively), However, the small number of transgenic embryos we obtained showed frequent CP (Table 2 and Table S1).

As CMV-driven *GFP* expression was variable and often difficult to visualize; and since we screen all embryos for phenotypic abnormalities, the utility of the *GFP* marker in these transposons is limited. Therefore, we removed the CMV-eGFP expression cassette to generate a smaller transposon containing only the U6-shRNA expression cassette (0.56 kb, Fig. 3E). Methylation by CpG methylases and reduced transposon size address two variables known to improve *SB* transposition efficiency [27] (Largaespada, D.A., personal communication). We performed multiple transgenic injections to investigate these variables and observed little difference in outcome (Table 2).

In contrast, utilization of a *PB* transposon that expresses a *Prdm16*-specific shRNA driven by the U6 Pol III promoter proved much more successful (Table 2 and Table S1). We compared the transgenic efficiency and phenotypic penetrance achieved utilizing differing amounts of the PB transposon plasmid DNA and *PBase* mRNA. Control experiments with no co-injected *PBase* mRNA produced yielded 59 embryos, 10 (17%) of which were transgenic with 1 (2%) exhibiting CP. Therefore, transgenic efficiency was within the normal range for a traditional transgenic DNA injection experiment, and the CP penetrance in transgenic embryos was 10%. The same amount of transposon DNA (∼2.0 µg/ml) co-injected with *PBase* mRNA (23 µg/ml) dramatically increased transgenic efficiency (68%), CP embryos (11%) and penetrance (16%). A substantial increase in *PBase* mRNA concentration (92 µg/ml) did not increase these values. On the contrary, we observed a marked increase in resorptions, decrease in live embryos and obtained only 10% (2/20) transgenic efficiency with only 1 affected embryo (5%). A slight reduction in *PB* transposon concentration (1.4 µg/ml) co-injected with lower *PBase* mRNA concentrations (17 and 23 µg/ml) yielded the most ideal conditions for these validation studies, increased transgenic efficiency (32% and 42%) and penetrance (29% and 27%) in 22 and 62 embryos, respectively. On average, use of 1.4–2.0 µg/ml PB transposon DNA and 17–23 µg/ml *PBase* mRNA resulted in 46% transgenic efficiency and 23% CP penetrance (Table 2).

To confirm *in vivo* knockdown of *Prdm16* in these embryos, nuclear protein lysates were isolated from two non-transgenic wild type and two transgenic CP embryonic heads harvested from a *PB* transposon plus *PBase* mRNA co-injection experiment. Relative PRDM16 protein levels were determined by Western analysis of these nuclear fractions to using a rabbit PRDM16-specific polyclonal antibody (Fig. 4I). Marked reduction of a pair of protein bands just over 150 kD in size in transgenic embryos with CP confirmed successful knockdown of *Prdm16*. Although the exact nature of these PRDM16 isoforms has not been determined, specific loss of these protein products has been demonstrated previously in mutant mice carrying *Prdm16* null alleles [21].

## Discussion

We chose to pursue transient transgenic RNAi knockdown during mouse embryogenesis as a means to rapidly validate loss of function gene mutations, which we have identified as part of an ENU mutagenesis screen for late embryonic phenotypic anomalies [15]. Transient transgenic RNAi knockdown has the obvious advantage of speed over standard homologous recombination in ES cells for rapid phenotypic validation or candidate gene screening. Mutant embryos deficient for expression of a gene of interest can be examined within 2–3 weeks of microinjection. Resources such as the RNAi Consortium (http://www.broadinstitute.org/rnai/trc) and RNAi Codex (http://cancan.cshl.edu/cgi-bin/Codex/Codex.cgi) increasingly facilitate the selection of gene-specific siRNA sequences to efficiently knock down gene function. Even with the selection of high-scoring siRNA target sequences predicted using bioinformatics tools, one must validate knockdown efficiency experimentally, which we did using a *luciferase*-based *in vitro* assay [26]. We examined a variety of vehicles for the delivery of gene-specific shRNAs into mouse embryos with the aim of producing transgenic mouse embryos with high frequency, which is crucial to such a screening strategy, especially given the potential variability of RNAi knockdown efficiency. All of these methods recapitulated the CP phenotype observed in *Prdm16* mutant mice. Lentivirus-infection and *PB* transposon-mediated transgenesis yielded the highest transgenic efficiency and phenotypic penetrance. Our studies were carried out using a single gene, *Prdm16*, to facilitate the comparative analysis of a multitude of shRNA delivery systems and variables; extension of these studies to additional candidate genes will be undertaken to validate the general application of our strategy.

Lentivirus infection has been used effectively to generate stable transgenic mammalian lines with both constitutive and conditional expression of transgenes and shRNAs [8], [9], [10], [31], [32]. This proved to be a viable strategy for transient transgenic RNAi knockdown in mouse embryos using *Prdm16*-specific shRNAs, although the specialized training and facilities necessary for working with these pathogens reduces its attractiveness as a universal tool for these studies.

The *SB* system is also a tractable means to perform *in vitro* and *in vivo* transgenic studies of many kinds, including cancer modeling, gene trapping, generation of transgenic mouse lines and insertional mutagenesis [33], [34], [35], [36]. Several factors have been shown to affect the transposition efficiency of *SB* transposons *in vitro*. There is a demonstrated decrease in transposition efficiency that is directly proportional to transposon size and *SB* transposase expression levels over a certain threshold (overproduction inhibition) [27], [37]. CpG methylation of *SB* transposons and heterochromatin formation has been shown to increase transposon excision from the genome and transposition of a plasmid into the genome, and *SB11* transposase shows a high affinity for heterochromatin [28], [29], [38]. Methylation of *SB* transposons has given rise to very high transgenic efficiency (up to 90%) in mice (Largaespada, D.A., personal communication). However, the heterochromatic state can potentially silence promoter activity, which would mitigate the advantage of increased transposition. We utilized methylated *SB* transposons containing CpG-free shRNA and *GFP* expression cassettes to attempt to achieve high transposition/transgenic efficiency without silencing shRNA and *GFP* expression. We tested many of the variables above in our SB transposon injections and achieved little increase in efficiency (Fig. 3A). We achieved very high transgenic efficiency using CpG-methylated CpG-free *SB* expression plasmids, but we did not obtain any fetuses that recapitulated the *csp1* mutant phenotype (Table S1). We examined transposon size and CpG methylation status via other CpG-containing *SB* transposons. Generally, these variations all resulted in low transgenic efficiency, but yielded a highly penetrant phenotype. Unfortunately, these attempts to optimize the *SB* transposition did not produce the high transposition/transgenic efficiency coupled with a high phenotypic penetrance that we desired. Certainly other variables could be adjusted, such as the amount of transposon DNA and *SB11* transposase mRNA injected, in order to improve this system; however, given our results using the *PB* transposon system, we did not pursue further optimization of the *SB* transposon system. Recently, a hyperactive *SB* transposase mutant (*SB100X*) with ∼100-fold increase in transposition efficiency over the first-generation *SB* transposase enzyme was reported [39]. Pronuclear injection of *SB100X* transposase and an *SB* transposon (CAGGS promoter driving *Venus* expression) into mouse embryos using amounts different than in our *SB* experiments resulted in 37% transgenic efficiency in newborn mice. Therefore, utilization of the *SB100X* transposase in combination with our *SB* shRNA-expressing transposons may improve transgenic efficiency to go along with the high phenotypic penetrance that we observed.

In contrast to our experience using *SB* transposition, *PB* transposon-mediated transgenesis yielded a substantial improvement in transgenic efficiency over traditional plasmid DNA injections and produced a highly penetrant phenotype. This result, combined with the observed reasonable phenotypic penetrance, makes *PB* transposition an attractive, nonviral approach to validate positionally-cloned gene mutations and screen candidate genes. A 4-5-fold increase of *PBase* mRNA levels resulted in more resorptions, less live embryos and low transgenic efficiency. Transposition efficiency is directly dependent upon increased transposase levels up to a certain threshold level [37]; therefore, our results may reflect this increased transposition efficiency and a corresponding deleterious effect on viability due to increased integration events with higher probability of disrupting essential genes and/or regulatory elements.

Clearly, additional modifications of this system can be considered. The addition of minimal mammalian insulator sequences flanking the shRNA expression cassette, such as the chicken hypersensitive site-4 (cHS4) chromatin insulator, may mitigate the potential negative effect on shRNA expression of methylation differences or position effects. One might also consider additional modifications to this system to more specifically examine loss of gene function during mouse embryogenesis, including gene-specific or temporally-specific RNAi transgenesis using mouse Pol II RNA polymerase or inducible promoter sequences.

In summary, we describe the use of transient transgenic RNAi knockdown to demonstrate the developmental consequences of a loss of function mutation. We carried out a detailed examination of the efficacy of lentivirus- and transposable element-based methods for the delivery of shRNA-expressing transgenes. Lentivirus infection and *PB* transgenesis achieved comparably favorable transgenic efficiency and phenotypic penetrance; however, the nonviral *PB* transposon system has significant advantages since no specialized training, equipment or facilities are required. Transient transgenic RNAi knockdown can be a universally tractable, rapid and powerful approach for use in human and mouse genetic studies to validate positionally cloned mutations and to screen candidate genes for developmental phenotypes.

## Materials and Methods

### shRNA selection and validation

*Prdm16*-specific shRNAs were chosen using informatics software that screened the *Prdm16* coding sequence for short 19-mer sequences meeting eight criteria for efficient knockdown of mRNA expression (score >7) described previously [10], [25]. Eight target sequences showing no homology to other mouse genes were selected for cloning into the pLenti-Lox3.7 (pLL3.7) lentivirus plasmid backbone and subsequent *in vitro* validation of knockdown efficiency (Table 1) [10]. RNAi knockdown efficiency was determined experimentally using a previously described *luciferase* reporter strategy [26]. RNAi target cDNA sequence contained in a Gateway Entry vector derived from a *Prdm16* EST clone, GenBank Accession No. CB248179.1 [21] was cloned into the 3′ UTR of a modified *Firefly luciferase* expression plasmid adapted for use as a destination vector in the Gateway cloning system (pGL3-DEST) via a LR clonase reaction to make the pGL3-DEST-*Prdm16* (Invitrogen). 100 ng pGL3-DEST-*Prdm16*, 25 ng pRL-tk (control *Renilla luciferase* expression plasmid) and 200 ng empty pLL3.7 or *Prdm16*-specific shRNA expression plasmid were transfected into 293T cells and incubated for 24–36 hours after which cells were lysed and *Firefly* and *Renilla* luciferase activity was measured as directed using the Dual-Luciferase Reporter Assay (Promega) in a Veritas Microplate Luminometer (Turner BioSystems). Relative luciferase activity values were calculated as the ratio of Firefly:Renilla luciferase in each transfected well, and each transfection was performed in duplicate. RNAi knockdown efficiency was taken as the ratio of the relative luciferase activity for the experimental shRNA plasmid over that for the pLL3.7 negative control transfection.

#### Antibodies and Western blotting

Affinity-purified PRDM16-specific antiserum raised against an N-terminal PRDM16 peptide was described previously [21]. Western blots were performed using established protocols. Nuclear fractions were isolated from embryonic heads as directed using the NE-PER Nuclear and Cytoplasmic Extraction kit (Pierce, Rockford, IL). Nuclear fractions (100 µg) were separated on a 6% polyacrylamide gel, transferred to PVDF membrane for 2 hours at 600 mA, and incubated in the presence of PRDM16 N-terminal (1∶7500) and Lamin B1 (1∶1000; Abcam) antisera, followed by antibody detection using the SuperSignal West Femto Maximum Sensitivity Substrate (Pierce).

### Lentiviral plasmid construction

Oligonucleotides containing the sense 19 nt RNAi target sequence followed by a short loop sequence and the reverse and complement 19 nt RNAi target sequence and poly-T terminator sequence. 60 pmoles of each oligonucleotide were annealed to make dsDNA in Annealing Buffer (100 mM Potassium Acetate, 30 mM Hepes-Potassium Hydroxide, pH 7.4 and 2 mM Magnesium Acetate) in a total volume of 50 µl using the following cycling conditions (95°C, 4 min., 70°C, 10 min. followed by incremental decrease (0.1°C/min.) to 4°C. Oligonucleotides were 5′-phosphorylated and designed with 5′ and 3′ overhangs to allow for directional cloning into XhoI/HpaI-digested, Calf Intestinal Phosphatase-treated pLL3.7 plasmid. Ligations were performed using 60 fmoles of annealed oligonucleotides and linearized plasmid in a 10 µl reaction volume using the Quick Ligation Kit (NEB) and transformed into *stbl3* chemically competent cells (Invitrogen). Transformants were screened by colony PCR using primers that flank the multiple cloning site of pLL3.7 (Table S2).

### *Sleeping Beauty* and *PiggyBac* transposon construction

#### Sleeping Beauty

Empty pKT2-HF transposon plasmid DNA and pT3TS-*SB11 transposase* expression plasmid were generously provided by David Largaespada (Univ. of Minn.). CpG-free plasmids pMOD-ZGFP::sh, pCpG-H1siRNA and pCpG-mcs (Invivogen) were used to generate CpG-free SB transposons to avoid gene silencing upon CpG methylation via SssI methylase (NEB). pKT2-HF and pCpG-H1siRNA plasmids were digested with EcoRI and HindIII restriction enzymes. The gel-purified H1siRNA expression cassette fragment was ligated into the digested and gel-purified pKT2-HF plasmid to make pKT2-HF-H1siRNA. A CpG-free synthetic GFP coding sequence was amplified from pMOD-ZGFP::sh plasmid DNA using oligonucleotides containing BglII (oBB1018) or NheI (oBB1019) restriction sites at their 5′ ends (Table S2). This PCR product and pCpG-mcs were digested with BglII and NheI restriction enzymes, gel-purified and ligated to make pCpG-GFP. pCpG-GFP and pKT2-HF-H1siRNA plasmids were each digested with EcoRI, and the fragment containing the GFP expression cassette was gel-purified and ligated into the digested pKT2-HF-H1siRNA plasmid to make pKT2-HF-GFP-H1siRNA. Alternative 5′ phosphorylated oligonucleotides with overhangs compatible with cloning into BbsI sites of the H1siRNA expression cassette (oBB1022-1033, Table 1) were annealed and ligated into BbsI-digested pKT-HF-GFP-H1siRNA as described previously for pLL3.7. In addition, GFP-minus transposons were created by ligation of annealed oligos into the BbsI sites of pKT2-HF-H1siRNA.

CpG-containing variations of these *SB* transposons were constructed by removing the CMV-eGFP; U6-shRNA expression cassettes from pLL3.7, pBB30, pBB31 and pBB33 from the pLL3.7 vector backbone by digestion with XbaI and EcoRI restriction enzymes. The pKT2-HF transposon plasmid was digested with SpeI and EcoRI, CIP-treated and ligated to the XbaI/EcoRI-digested CMV-eGFP; U6-shRNA expression cassettes. The empty plasmid backbone is referred to as pKT2-HF-U6-shRNA-GFP. Later these plasmids were digested with HindIII to remove the CMV-eGFP expression cassette to make pKT2-HF-U6-shRNA.

#### PiggyBac

Empty *PB* transposon plasmid DNA (pCyL50) and pCMV-*PBase* expression plasmids were generously provided by Pentau Liu (Sanger). The U6-shRNA cassettes from pBB30 were amplified using primers oBB1336/1337 (504 bp) that contained AscI or PacI restriction sites at their 5′ ends. These PCR products and pCyL50 plasmid DNA were digested with AscI/PacI and ligated together to make control and *Prdm16*-specific shRNA-expressing *PB* transposons (Table 1 and Table S2). PvuII digestion excised the IR-U6-nt622shRNA-IR fragment to be used for transgenesis. The *PB* transposase plasmid used as template for *in vitro* transcription reactions was constructed as follows. Empty pT3TS plasmid backbone was obtained by digesting pT3TS-*SB11* with BglII [40]. The oligonucleotide linker primers oBB1443 and oBB1444 (HindIII-NheI-XbaI) designed to have BglII-compatible 5′ ends at each end were annealed as described previously and ligated to BglII-digested, CIP-treated pT3TS to make pT3TS-linker (pBB231). Colonies were screened for orientation of the linker by colony PCR using T3/oBB1444. The *PB* transposase coding sequence was amplified from pCMV-*PBase* plasmid DNA using oBB1445 and oBB1437, which were designed with HindIII restriction site, Xenopus Globin 5′ UTR and BglII, NdeI, SacII and NheI restriction sites or an SpeI restriction site at their 5′ ends, respectively. This PCR product and pBB231 plasmid DNA were digested with HindIII/SpeI and ligated together to make pT3TS-*PBase* (pBB232).

Linearized and gel purified (Qiagen) pT3TS-*SB11* (BamHI) or pT3TS-*PBase* (XbaI) plasmids were used as template to make 5′-capped *SB11* or *PBase* mRNA, respectively, using the mMessage mMachine High Yield Capped mRNA T3 Transcription Kit (Ambion). mRNA was purified using NucAway Spin columns (Ambion). After determining RNA concentration, samples were aliquoted in 5 µl volumes and stored at −80°C.

### Transgenic mice

*Prdm16* shRNA containing *SB* and *PB* transposon plasmids were digested with PvuII (*SB*) or PvuII/BspHI (*PB*) to linearize or remove the plasmid backbone, respectively. Transposon fragments were purified using either the UltraClean GelSpin DNA purification kit (Mol Bio, Carlsbad, CA) or electroelution followed by concentration using the Wizard® DNA Clean-Up System (Promega). Transgenic mice were produced by transgenic injection of each shRNA-expressing transposon plasmid construct along with *in vitro* transcribed 5′-capped *SB11* or *PB* transposase mRNA into the pronuclei of fertilized eggs [41]. For *SB* transposon injections plasmid DNA was diluted to 4 µg/ml in injection buffer (5 mM Tris-Cl pH 7.5, 0.1 mM EDTA), and *SB* mRNA is added to a concentration of 10 µg/ml, aliquoted and stored at −70°C about 2–3 days before injection. For *PB* transposon injections plasmid DNA was diluted to 1.4–2.0 µg/ml of DNA along with 17, 23, 92 µg/ml or no *PBase* mRNA in injection buffer. CD-1 females were used as recipients for injected embryos.

All mice were housed in a 12-h light, 12-h dark cycle with unlimited access to tap water and Purina 5008 or 5020 chows. All procedures using mice were approved by the University of Michigan Committee on Use and Care of Animals, and all experiments were conducted in accordance with the principles and procedures outlined in the NIH Guidelines for the Care and Use of Experimental Animals.

Foster mother mice were euthanized on E15.5, E16.5 or E17.5 to screen potential transgenic embryos for cleft palate. For each embryo assayed the limbs and tail were collected for genotyping, the head was fixed in 3.7% Formaldehyde in Phosphate Buffered Saline, pH 7 overnight at 4°C, and, for select embryos, the body was stored in RNA Later (Ambion) at −20 C. After fixing, heads were washed and dehydrated through a graded ethanol series to 70% ethanol and stored at −20°C. Genotyping samples were processed as described previously [42]. All embryos were genotyped for the presence of a transgene by PCR using oligonucleotide primers provided in Table S2.

## Supporting Information

Table S1 Detailed summary of transgenic RNAi injections.
(0.11 MB DOC)

Table S2 Additional oligonucleotide primers.
(0.06 MB DOC)
