# Supplementary material for: A Transient Transgenic RNAi Strategy for Rapid Characterization of Gene Function during Embryonic Development
Source: PLoS One. 2010 Dec 16;5(12):e14375. doi: 10.1371/journal.pone.0014375 (PMC3002952; doi:10.1371/journal.pone.0014375)
Supplement: Table S1 — Detailed summary of transgenic RNAi injections. (0.11 MB DOC) [file pone.0014375.s001.doc]

Supplementary Table 2. Detailed summary of transgenic RNAi injections

| Construct | Methylation | Embryos injected | Resorptions | # Embryos | TG | CP | TG Frequency | CP Frequency | TG w/ CP Frequency | CP, not TG | Frequency live embryos | Freqency resorptions |
| --- | --- | --- | --- | --- | --- | --- | --- | --- | --- | --- | --- | --- |
| Lentivirus plasmid control | No | ND | ND | 88 | 16 | 0 | 0.18 | 0 | NA | 0 | ND | ND |
| U6; *GFP* |  |  |  |  |  |  |  |  |  |  |  |  |
| Lentivirus plasmids; nt1266, nt1894 | No | ND | 7 | 248 | 8 | 0* | 0.03 | 0* | 0 (2 TG arrested at E11.5) | 0 | ND | ND |
| U6; *GFP* |  |  |  |  |  |  |  |  |  |  |  |  |
| lentivirus; nt622 | No | ND | 2 | 55 | 26 | 5* | 0.47 | 0.09 | 0.15 | 1 | ND | ND |
| U6; *GFP* |  |  |  |  |  |  |  |  |  |  |  |  |
| *SB*; nt622 scrambled | Yes | ND | 21 | 56 | 32 | 0 | 0.57 | 0 | 0 | 0 | ND | ND |
| CpG-free H1; *GFP* |  |  |  |  |  |  |  |  |  |  |  |  |
| *SB*; nt622 | Yes | ND | 6 | 79 | 54 | 0 | 0.68 | 0 | 0 | 0 | ND | ND |
| CpG-free H1; *GFP* |  |  |  |  |  |  |  |  |  |  |  |  |
| *SB* control | No | ND | 5 | 32 | 4 | 0 | 0.13 | 0 | 0 | 0 | ND | ND |
| U6; *GFP* |  |  |  |  |  |  |  |  |  |  |  |  |
| *SB*; nt1266, nt1894 | No | ND | 89 | 108 | 5 | 4 | 0.05 | 0.04 | 0.8 | 0 | ND | ND |
| U6; *GFP* |  |  |  |  |  |  |  |  |  |  |  |  |
| *SB*; nt1266 | No | ND | 24 | 57 | 4 | 3* | 0.07 | 0.05 | 0.5* | 1 | ND | ND |
| U6; no *GFP* |  |  |  |  |  |  |  |  |  |  |  |  |
| *SB*; nt1266 | SssI | ND | 81 | 73 | 12 | 2 | 0.16^$^ | 0.03 | 0.17^$^ | 0 | ND | ND |
| U6; no *GFP* |  |  |  |  |  |  |  |  |  |  |  |  |
| *PB*; nt622 (~2.0 μg/ml) | No | 156 | 28 | 59 | 10 | 1 | 0.17 | 0.02 | 0.10 | 0 | **0.38** | 0.18 |
| U6: no *GFP* |  |  |  |  |  |  |  |  |  |  |  |  |
| No *PBase* |  |  |  |  |  |  |  |  |  |  |  |  |
| *PB*; nt622 (~1.4 μg/ml) | No | 108 | ND | 22 | 7 | 2 | 0.32 | 0.09 | 0.29 | 0 | 0.20 | ND |
| U6: no *GFP* |  |  |  |  |  |  |  |  |  |  |  |  |
| *PBase* (17 μg/ml) |  |  |  |  |  |  |  |  |  |  |  |  |
| *PB*; nt622 (~1.4 μg/ml) | No | 225 | 33 | 62 | 26 | 7 | 0.42 | 0.11 | 0.27 | 0 | 0.28 | 0.15 |
| U6: no *GFP* |  |  |  |  |  |  |  |  |  |  |  |  |
| *PBase* (23 μg/ml) |  |  |  |  |  |  |  |  |  |  |  |  |
| *PB*; nt622 (~2.0 μg/ml) | No | 192 | 28 | 28 | 19 | 3 | 0.68 | 0.11 | 0.16 | 0 | 0.15 | 0.15 |
| U6: no *GFP* |  |  |  |  |  |  |  |  |  |  |  |  |
| *PBase* (23 μg/ml) |  |  |  |  |  |  |  |  |  |  |  |  |
| *PB*; nt622 (~1.4 μg/ml) | No | 227 | 39 | 20 | 2 | 1 | 0.10 | 0.05 | 0.50 | 0 | **0.09** | 0.17 |
| U6: no *GFP* |  |  |  |  |  |  |  |  |  |  |  |  |
| *PBase* (92 μg/ml) |  |  |  |  |  |  |  |  |  |  |  |  |

*Denotes one or more transgenic embryos showing early embryonic growth arrest prior to palate fusion.

^$^Three of 24 resorptions were transgenic.
