# Supplementary material for: A Transient Transgenic RNAi Strategy for Rapid Characterization of Gene Function during Embryonic Development
Source: PLoS One. 2010 Dec 16;5(12):e14375. doi: 10.1371/journal.pone.0014375 (PMC3002952; doi:10.1371/journal.pone.0014375)
Supplement: Table S2 — Additional oligonucleotide primers. (0.06 MB DOC) [file pone.0014375.s002.doc]

Supplementary Table 3. Additional oligonucleotide primers

| Primer # | Primer | Sequence (5’-3’) | Product Size (bp) |
| --- | --- | --- | --- |
| 654 | pLL3.7test-F | GCACAGACTTGTGGGAGAAG | 392 (correct) vs 336 (empty) |
| 655 | pLL3.7test-R | CCTGCTGGAATCTCGTGAA | 392 (correct) vs 336 (empty) |
| 1018 | CpGfreeGFP-BglII-F | AGTTCTAGATCTATGGTTTCTAAGGGAGAAGAACTCT | 700 |
| 1019 | CpGfreeGFP-BglII-F | CTGTTCGCTAGCTCCCAGAGTAATTCCTGCTGC | 700 |
| 1032 | CpGfree-test-F | TGTGTTCTGGGAAATCACCAT | 210 (correct) vs 520 (empty) |
| 1033 | CpGfree-test-R | TTATTTCTTTCATCACATTCCCAGT | 210 (correct) vs 520 |
| 1336 | AscI-U6-shRNA-F | GATTGGCGCGCCGCTCTAGAGATCCGACGCCGCCAT | 504 |
| 1337 | PacI-U6-shRNA-R | GCATATTAATTAAATGGCGGTAATACGGTTATCCACG | 504 |
|  | SB-CpGfree-F | TTTCCCATTATTGGCACATACA | 382 |
|  | SB-CpGfree-R | GAGTTCTTCTCCCTTAGAAACCA | 382 |
|  | SB-shRNA-GFP-F | CGTGTACGGTGGGAGGTCTA | 479 |
|  | SB-shRNA-GFP-R | GTCCTCCTTGAAGTCGATGC | 479 |
|  | SB-shRNA-F | ATCACATTCCCAGTGGGTCA | 150 |
|  | SB-shRNA-R | AGGGGAGTAGCCGAGCTT | 150 |
| 1493 | PB-shRNA-F | AAATGGGTAGTTCTTTAGACGATGAG | 155 |
| 1494 | PB-shRNA-R | CGAGCTTCTCCCACAAGTCT | 155 |
| 1443 | pT3TS-Ad-S | 5'PHOS-GATCTAAGCTTGCTAGCTCTAGA-3' | NA |
| 1444 | pT3TS-Ad-AS | 5'PHOS-GATCTCTAGAGCTAGCAAGCTTA-3' | NA |
| 1445 | PBase-ORF-F | GATAAGCTTGCTTGTTCTTTTTGCAGAAGCTCAGAATAAACGCTCAACTTTGGCAGATCTCATATGTCCGCGGGCTAGCCACCATGGGCAGCAGCCT | 1794 |
| 1437 | PBase-ORF-R | GTCAATACTAGTGCTCATCAGAAACAGCTCTGGCA | 1794 |
